# Registry of Births and Deaths in Hyde Park

**Published:** 1878-10

**Authors:** J. Ramsey Flood

**Affiliations:** Health Officer, Hyde Park


HEALTH OFFICE, HYDE PARK, Aug. 23, 1878.

*Editor Chicago Medical Journal and Examiner:*

The following sections of a revised ordinance, passed by the Board of Trustees of Hyde Park, and which went into effect on the 27th day of August, 1878, will be of interest to the physicians of Chicago and vicinity, and for that reason I send them to you for insertion in the JOURNAL, as the most convenient and sure way of reaching them.

"SECTION 10. Every physician, midwife, or other person who may professionally assist or advise at any birth or death, shall make a return of such birth or death to the health department of this village, such as now required by the State Board of Health to the county clerk. Said returns of births shall be made to said health department within four days after the date of such birth, and returns of deaths shall be made to said health department within twenty-four hours after such death may occur. It shall be the duty of said village physician to transmit the returns of births and deaths aforesaid to the county clerk within the time now prescribed by law.

"Section 11. No remains of any human being, having died within the corporate limits of the village, or whose body may be found within the limits of the same, shall be removed from this village for interment or otherwise, nor shall said remains be interred within the corporate limits of Hyde Park, until the certificate, as required in section 10 of this chapter, shall have been filed at the office of the health department, and a permit first had and obtained for such removal or interment."

It will be seen, by a noncompliance on the part of attending physicians upon their late patients, that the friends of deceased will be put to no inconsiderable trouble before interment can be had, as no interment can be obtained within Hyde Park of such deaths as may occur within the limits of the same until a *burial permit* be had, and no such permit can be had except on the death certificate of the attending physician, or the finding of a coroner's jury, as the case may require.

If physicians whose patients die within the limits of Hyde Park, and whose friends wish to inter them at Oakwood Cemetery, will please bear this in mind, they will save the friends of the deceased much trouble by promptly making returns of such death, as required above.

J. Ramsey Flood,
*Health Officer, Hyde Park.*

---

In the *White Mountain Echo*, of Sept. 7, 1878, we find recorded the proceedings of the United States Hay Fever Association, convened at Bethlehem, N. H., in the very heart of the White Mountains. The perusal of these pages is sufficient to move any one to—a sneeze. "O, ye tears,"—but stay—this is the month of October.

Phimosis as a Cause of Hernia.—In the examination of 51 children affected with congenital phimosis, Kempe discovered that 31 had hernia; five, double inguinal; a few, umblical. None suffered at birth, and the tighter the preputial stenosis, the earlier the hernia. All were relieved by circumcision; in five, the latter operation relieved the hernia.—(*Lancet, July*, 1878.)

